# Changes in Parenteral Nutrition Requirements and BMI in Patients with Parenteral Nutrition-Dependent Short Bowel Syndrome after Stopping Teduglutide—9 Years of Follow-Up

**DOI:** 10.3390/nu14081634

**Published:** 2022-04-14

**Authors:** Zuzanna Zaczek, Paulina Jurczak-Kobus, Mariusz Panczyk, Joanna Braszczyńska-Sochacka, Krystyna Majewska, Marek Kunecki, Karolina Dąbrowska, Jacek Sobocki

**Affiliations:** 1 Department of Human Nutrition, Faculty of Health Sciences, Medical University of Warsaw, Erazma Ciołka 27, 01-445 Warsaw, Poland; 2 Department of General Surgery and Clinical Nutrition, Centre of Postgraduate Medical Education, Czerniakowska 231, 00-416 Warsaw, Poland; paulina_jurczak@wp.pl (P.J.-K.); krysiamajewska@wp.pl (K.M.); k.lachowicz89@gmail.com (K.D.); jsobocki@mp.pl (J.S.); 3 Department of Education and Research in Health Sciences, Faculty of Health Sciences, Medical University of Warsaw, Litewska 14/16, 00-581 Warsaw, Poland; mariusz.panczyk@wum.edu.pl; 4 Clinical Nutrition Department, M. Pirogov Hospital, Wólczańska 191/195, 90-531 Lodz, Poland; joanna.braszczynska@gmail.com (J.B.-S.); marek.kunecki@vp.pl (M.K.)

**Keywords:** short bowel syndrome (SBS), teduglutide, home parenteral nutrition (HPN), GLP-2, follow-up

## Abstract

Teduglutide (TED) is widely used in patients with short-bowel-syndrome-associated intestinal failure (SBS-IF) to enhance intestinal adaptation and reduce the need for parenteral support (PS). There are limited data on the effects of discontinuing TED. In this study, we describe the changes in parenteral nutrition (PN) requirements and body mass index (BMI) in a 9-year follow-up of patients receiving home parenteral nutrition after discontinuation of the TED treatment. We performed a retrospective analysis of changes in weekly PN orders and BMI in all patients with PN-dependent SBS from two Polish home parenteral nutrition (HPN) centers who received teduglutide between 2009 and 2013 and still required HPN 9 years after discontinuation of the TED treatment. Data included in the analysis were collected prospectively at mandatory visits to the HPN centers at 12, 24, 60, 84, and 108 months after drug discontinuation and compared with values before and after TED treatment. Weekly PN volume values varied significantly between all of the above time points from baseline to 9 years after TED discontinuation (χ^2^ = 34.860, *p* < 0.001). After an initial increase within the first year after treatment discontinuation (not statistically significant), the PN volume requirements remained stable for 4 years and increased 5–9 years after treatment discontinuation. The rate of patients requiring an increase in PN volume was 84.62% at 60 and 84 months and 92.30% at 108 months. At 9 years after cessation of the TED treatment, 53.85% of the study group required a 21.21% increase in PN volume compared with values before treatment. The need for PN volume in patients with PN-dependent SBS who discontinued the TED treatment increased within the first year and 4–5 years after treatment cessation, and in some cases might even exceed pretreatment values after 9 years.

## 1. Introduction

Short bowel syndrome (SBS) is a condition in which absorption of nutrients, fluids, and electrolytes is impaired due to intestinal resection, congenital anomaly, or less commonly as a result of small bowel dysfunction [1]. The spectrum of malabsorption ranges from intestinal insufficiency to intestinal failure and depends on the length and anatomy of the remaining small bowel, among other factors [2]. SBS is considered the most common pathophysiological mechanism for chronic intestinal failure (IF) in the adult population and accounts for approximately two-thirds of adult patients receiving home parenteral nutrition (HPN) [3,4,5]. Long-term total or supplemental parenteral support (PS), consisting of parenteral nutrition (PN) and/or intravenous fluids (IVF), usually in the form of HPN, is often required to maintain fluid and electrolyte balance and to meet the nutritional needs of SBS-IF patients. The need for intravenous nutritional supplementation depends on the type of SBS and severity of malabsorption [6,7]. PN is a life-saving primary therapy for SBS patients that has been shown to be safe. However, it also carries the risk of complications, including hepatic and renal dysfunction, as well as complications resulting from central venous catheter (CVC) maintenance, such as catheter-related bloodstream infections (CRBSI) or thrombosis [8,9]. Therefore, finding ways to improve the absorptive capacity of the remnant bowel and reduce PS is crucial to provide long-term well-being for those patients.

Intestinal adaptation is a natural process that primarily occurs in the first two years following resection, especially in patients with colon in continuity [10], and leads to improved absorption and thus the possibility of partial or complete weaning from PN [11,12]. Numerous studies over the last two decades have shown that gastrointestinal hormones play an important role in the process of postresective intestinal rehabilitation [13,14,15]. The strongest evidence supports the efficacy of glucagon-like peptide 2 (GLP-2), an endogenous hormone secreted by L-cells in the ileum and colon, that promotes villus height and crypt depth and prolongs gastric emptying [16,17,18]. 

Teduglutide (TED), a recombinant analog of GLP-2, is a novel drug approved by the U.S. Food and Drug Administration (FDA) in 2012 for the treatment of patients with PN-dependent SBS [19,20]. The efficacy of teduglutide in enhancing gut adaptation in adult patients, leading to a reduction in parenteral support, was demonstrated by Jeppesen et. al. [21,22] in phase III clinical trials. Since then, it has also been reported in several real-life studies [23,24,25,26]. In the consensus of the Polish Intestinal Failure Centres [27], experts concluded that there were two groups of patients with HPN for whom the treatment with teduglutide could be beneficial: those with the possibility of complete weaning from PN and those with a poor prognosis for whom treatment would be life-saving. 

Although teduglutide is currently approved in Europe and Canada as well as in the United States, it is still very difficult to access because it is not reimbursed in many countries and the therapy is very expensive (>400,000 USD/year) [28,29]. As a result, treatment is often discontinued after completion of the clinical trial. It seems that short duration of action is a weak point of this GLP-2 analog, as its effect diminishes rapidly after discontinuation. Although the safety and efficacy of teduglutide is a subject of an increasing number of studies, there are few studies on the clinical outcomes and PN dependence of patients who discontinued treatment. The aim of this study was to assess the changes in PN orders and body mass index (BMI) in HPN patients during a 9-year follow-up period after they stopped taking teduglutide. To the best of the authors’ knowledge, this is the first study to provide data over such a long follow-up period, approximately a decade.

## 2. Materials and Methods

### 2.1. Patient Population

This real-life multicenter study was designed as a retrospective analysis of prospectively collected medical data of patients with PN-dependent SBS after cessation of teduglutide treatment. Patients received home parenteral support both before and after TED treatment at two national reference HPN centers in Poland. We included all patients who received teduglutide between 2009 and 2013 in a randomized, placebo-controlled multicenter trial (NCT00798967) followed by a 2-year extension study (NCT00930644), and still required HPN 9 years after discontinuation of teduglutide therapy. Patients who were weaned from HPN or died during the observation period were excluded from the analysis.

### 2.2. Data Collection

Data were collected and documented prospectively during mandatory quarterly visits in HPN centers. The visits, conducted by an interdisciplinary team of specialists, included patient interview, blood tests, nutritional assessment, medical examination, and parenteral nutrition formula adjustment (if needed). Nutritional assessment was performed by qualified dietitians or nurses and included anthropometric measurements of height and body weight. Body weight was measured with an electric scale (Fawag S.S. model ZOL-3.4. w. WTL, Lublin, Poland) with an accuracy of 0.1 kg, according to the recommendations of the Centers for Disease Control and Prevention (CDC) [30]. Blood tests included blood morphology, coagulation factors (prothrombin time, PT and international normalized ratio, INR), glucose, total cholesterol, triglycerides, venous blood gases, venous ionogram (sodium, potassium, calcium, chlorides, magnesium, phosphorus), total serum protein, serum albumin, urea, creatinine, C-reactive protein, and liver function parameters including aspartate aminotransferase (AST), alanine aminotransferase (ALT), alkaline phosphatase (ALP), lactate dehydrogenase (LD), bilirubin, and gamma-glutamyl transferase (GGT). Any adjustments to parenteral nutrition prescriptions were made by a physician based on the results of all the above parameters. All data collected during visits were kept in the patient’s file. 

### 2.3. Study Design and Data Analysis

**Figure 1:**
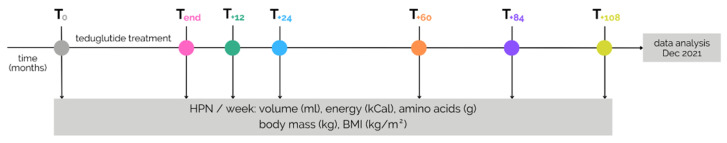
Study design and timeline.

A retrospective analysis of data obtained during visits was performed in December 2021 and included the following parameters: body weight, BMI, and weekly PN volume (ml), energy (kCal) and amino acids (g) with PN volume as the primary outcome variable. BMI was calculated by dividing body weight (kg) by height (m) squared. The aforementioned parameters were assessed at seven time points: before initiation of the TED treatment (after optimization of PN orders) (T_0_), after cessation of treatment (T_end_), and 12, 24, 60, 84, and 108 months (1, 2, 5, 7, and 9 years) after discontinuation of the drug. The detailed study timeline is shown in Figure 1. The study was approved by the Ethics Committee of Centre of Postgraduate Medical Education (protocol code 96/2021, date of approval 10 November 2021). To protect individuals’ information, all data were anonymized.

### 2.4. Statistical Analysis

Selected clinical variables were presented using descriptive statistics: median (Mdn) and semi-interquartile range (IQR/2), coefficient of variation (CV) and range (Min-Max). Null hypothesis testing was calculated using nonparametric tests: a Mann–Whitney U test with continuity correction (for independent samples) and a Wilcoxon signed-rank test or Friedman test with post hoc Dunn–Sidàk test (for dependent samples). It was assumed that the small sample size and the characteristic of the distribution of the dependent variable prevented the correct use of the parametric test. All statistical calculations were carried out with STATISTICATM version 13.3 package (TIBCO Software Inc., Palo Alto, CA, USA). The statistical null hypothesis was rejected if the two-tailed *p* < 0.05.

## 3. Results

### 3.1. Group Characteristics

A total of 13 patients from two HPN centers met the eligibility criteria and were included in this study. The underlying conditions for SBS were as follows: intestinal ischemia 23.1% (*n* = 3), volvulus 15.4% (*n* = 2), trauma 15.4% (*n* = 2), surgical complications 15.4% (*n* = 2), Crohn’s disease 7.7% (*n* = 1), ulcerative colitis 7.7% (*n* = 1), radiation enteritis 7.7% (*n* = 1), and Hirschsprung disease 7.7% (*n* = 1). A colon in continuity was present in 84.62% (*n* = 11) of patients, of which seven had between 50% and 75% of the colon length, three patients had the entire colon, and one patient had approximately 20% of the colon. The distribution of SBS types in patients with a colon was as follows: jejunoileal anastomosis (SBS type II) 76.92% (*n* = 10) and jejunocolic anastomosis (SBS type III) 7.69% (*n* = 1). There were two patients (15.38%; one male and one female) with no colon in continuity and with an end jejunostomy (SBS type I). Detailed characteristics of the study group, including the data on duration of HPN and treatment with teduglutide are shown in Table 1. 

#### Predrug Values and Drug Response

Based on the definition of a ≥20% reduction in PN volume during the TED treatment, all patients in the study group were drug responders, as the reduction in weekly PN volume ranged from 30 to 57% (median 46%). Values for body weight, BMI, and weekly PN requirements before (T_0_) and after treatment (T_end_) are shown in Table 2.

### 3.2. Anthropometric Measures

**Figure 2:**
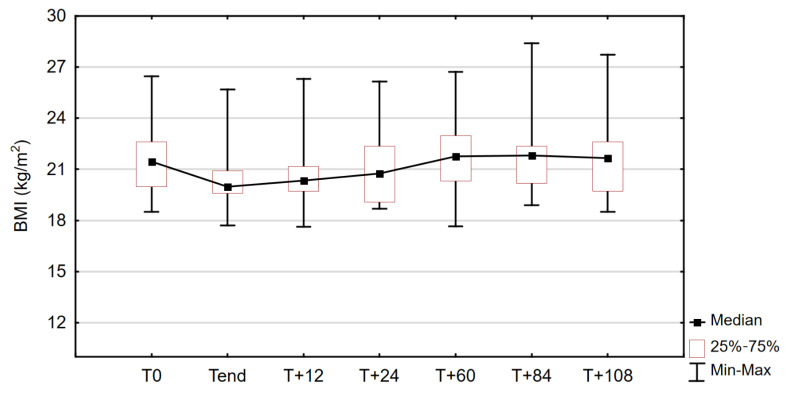
Changes in body mass index (BMI) in the study group throughout the observation period; Friedman test: χ^2^ = 23.448, *p* < 0.001.

Body weight, reflected by body mass index, varied significantly between the time points studied. The data on the change in BMI for the study sample can be found in Figure 2.

Compared with T_end_, significant differences in body weight (as measured by BMI) were found only at 60 and 84 months (5 and 7 years) after the end of the TED treatment. At T_+60_, the median body weight in the study group was 54 kg and was significantly higher than T_end_ (*p* = 0.049), as it increased by an average of 4.85 ± 3.71 kg in 84.62% of patients (*n* = 11). After another 2 years (T_+84_), the body weight was still significantly higher in the study sample compared to post-treatment measurements (*p* = 0.007). 

**Figure 3:**
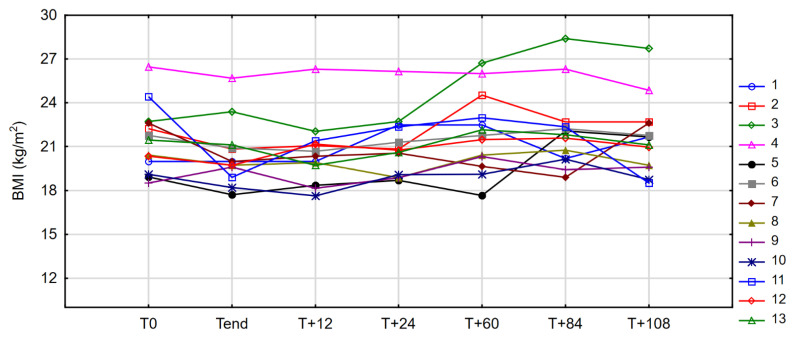
Changes in body mass index (BMI) in individual patients between observation time points (case profiles).

Compared with pretreatment values, no significant differences were observed at any of the time points studied. At the endpoint of observation (T_+108_), the median body weight was virtually the same as at baseline (54.7 vs. 55.0 kg), and in three patients, the body weight was identical to T_0_ values. The changes in BMI of each patient during the entire observation period, including baseline and post-treatment values, are shown in Figure 3.

### 3.3. Parenteral Nutrition Requirements

#### 3.3.1. PN Volume

Data on weekly PN volume 12, 24, 60, 84 and 108 months (1, 2, 5, 7, and 9 years) after cessation of the TED treatment are summarized in Table 3.

**Figure 4:**
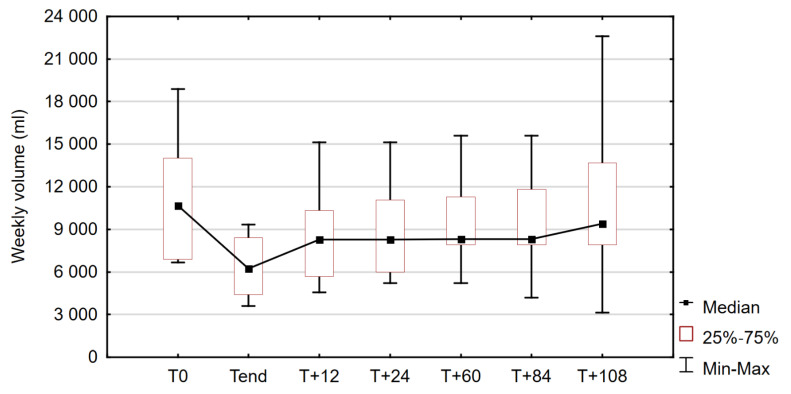
Comparison of weekly volumes of parenteral nutrition (PN) before and after treatment with teduglutide (TED) and 12, 24, 60, 84, and 108 months later; Friedman test: χ^2^ = 34.860, *p* < 0.001.

The values varied significantly between observation time points. Changes in median weekly PN volume in the study group during the observation period are shown in Figure 4.

At 12 months (1 year) after discontinuation of TED (T_+12_), weekly volumes of parenteral nutrition were increased by 13.9–101.6% (median 44.4%) in 69.3% of patients (*n* = 9) compared with post-treatment values (T_end_), corresponding to an increase of 1000–6720 mL (median: 1670) per 7 days. In three patients (23%), the PN volume remained unchanged and in one case a decrease of 1500 mL was observed. In one patient, the weekly volume at T_+12_ exceeded baseline (T_0_) by 1820 mL, but the mean values at T_+12_ were still statistically significantly lower in the study group than before administration of TED (*p* = 0.027). 

After another year (T_+24_), the weekly volume orders were higher compared to T_end_ in 84.62% of the study group (*n* = 11), by 47.72%. The PN requirements of one of the patients with stable (male, SBS type I) and one patient with decreased (male, SBS type III) volume in the first year remained unchanged at this point of the study, and in five other cases (38.46%) the values did not change in the second year after discontinuation of TED. No statistical significance was found when the PN values at T_+24_ were compared with both pretreatment and post-treatment values. 

At the time when 80 months (5 years) had passed since the end of the drug administration (T_+60_), the mean need for PN volume in the study group was significantly higher (*p* = 0.036) compared to the T_end_ values, but there was no statistical significance compared to baseline (T_0_). A further increase with respect to T_+24_ was observed in four cases, while in the remaining patients (69.23%), the PN volumes had not changed in the previous 3 years. In three patients, T_+60_ orders exceeded baseline values by 1405, 1435, and 2710 mL, representing increases of 20.36, 20.92, and 40.51%, respectively. 

At T_+84_, 84 months (7 years) after completion of the TED treatment, we observed changes in PN volume in 46.15% of patients (*n* = 6) compared with the previous measurement (T_+60_), and an increase in PN volume was observed in four of them. In the patient whose PN requirement decreased at T_+12_ and then remained stable for 6 years, a further decrease of 1800 mL was observed, so that the volume was 44% and 60.67% lower at T_+84_ compared with T_end_ and T_0_, respectively. In 11 patients (84.62% of the group), the weekly PN volume was higher than after treatment, as reflected by a median increase of 64.01% (4319 mL). The weekly volume of intravenous supply in the one patient whose HPN prescription was stable since the end of the TED treatment remained unchanged. The differences in mean PN volume between T_+84_ and T_end_ for the study group were statistically significant (*p* = 0.007). At this time point, PN volume was higher than pretreatment in four cases, as baseline values were exceeded in one additional patient (compared to T_+60_). In one case, the T_+84_ and baseline values coincided.

At the endpoint of this study, 108 months (9 years) after discontinuation of TED (T_+108_), a significant difference in weekly PN volume was observed compared with the values achieved during treatment (T_end_) (*p* < 0.001). Post-treatment measures were exceeded in 92.30% of the patients studied (*n* = 12) and the increase ranged from 26.76 to 169.17% (median 76.06%) with the highest results in two patients with SBS type II and over 50% of colon in continuity. In the patient with end jejunostomy, who did not require PN volume adjustment up to this point, an addition of 10,105 mL per 7 days was required at T_+108_, representing an increase of 122.19% compared to T_end_ and 31.11% with respect to baseline values. In one case (a patient whose PN volume decreased or remained stable throughout the observation period), the T_+108_ values were 58% lower than the T_end_ values, after a further reduction of 1050 mL between T_+84_ and T_+108_. Compared with baseline PN requirements, an increase ranging from 0.56 to 44.84% (median 21.21%) was observed in seven patients, who constituted more than half of the study group. In the remaining six patients the median T_+108_ PN volume was lower than T_0_ by 26.19%. 

**Figure 5:**
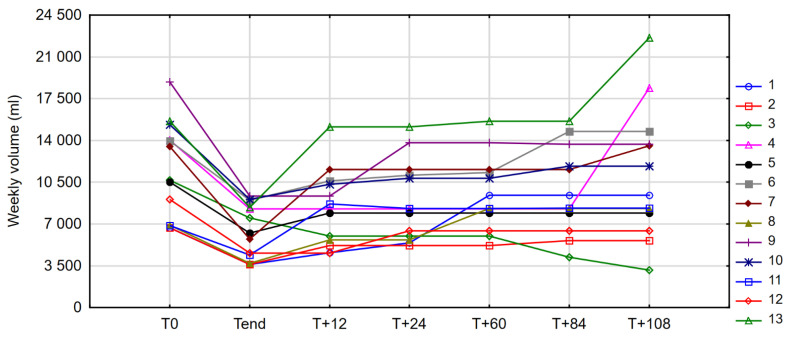
Changes in weekly parenteral nutrition (PN) volume in individual patients between observation time points (case profiles).

The case profiles of changes in weekly PN volume throughout the observation period are presented in Figure 5.

#### 3.3.2. PN Energy and Amino Acid Content

Comparing all time points studied from baseline to T_+108_, significant differences were found in weekly values of energy (χ^2^ = 34.39936, *p* < 0.001) and protein content (χ^2^ = 22.67442, *p* < 0.001). Measurements of weekly PN energy and amino acids at 12, 24, 60, 84, and 108 months (1, 2, 5, 7, and 9 years) after completion of the teduglutide treatment are summarized in Table 4. The differences in the above two parameters compared with T_0_ were not statistically significant. The mean PN energy requirements remained stable in the first 24 months (2 years) after cessation of the TED therapy and significantly increased 60, 84, and 108 months (5, 7, and 9 years) after teduglutide discontinuation (*p*-values: 0.012, 0.02, <0.001, respectively) compared to T_end_ values. The differences in PN amino acid content compared to T_end_ were not statistically significant at any of the time points studied.

## 4. Discussion

The aim of this study was to investigate changes in body weight and need for parenteral support (with PS volume as the primary outcome variable) in patients with SBS who received teduglutide injections for 25–37 months, discontinued treatment, but did not achieve enteral autonomy, and still required HPN 9 years later. To the author’s knowledge, this study is the first to present real-life data on the effects of TED treatment discontinuation over such a long period of time. We reported that the values of body weight (reflected by BMI), weekly PN volume, and energy and amino acid content of parenteral nutrition admixture were statistically significantly different between the time points studied. An upward trend was observed in all parenteral nutrition composition parameters analyzed. The results of the post hoc analysis showed that patients required statistically significantly higher PN volume and PN energy content 60, 84, and 108 months (5, 7, and 9 years) after stopping teduglutide. It is important to point out that the increase in PN energy and protein content was followed by an increase in body mass. This suggests that even a lower macronutrient content may have been sufficient to maintain a stable body weight.

There are limited data on what to expect after cessation of teduglutide treatment in patients with SBS-IF receiving home parenteral nutrition. Regarding the effects on anthropometric measures and PN requirements, the only available results are from Compher et al. [31]. The authors described changes in PS weekly volume and BMI over a 12-month (1 year) period after discontinuation of TED in patients who completed the phase III trial of teduglutide and in a subset of drug responders. In the present study, 69.3% (*n* = 9) of patients required an increase in PN volume of 1.67 L (median) per week 12 months after stopping TED, and a further decrease was reported in one case (7.69%). In comparison, Compher et al. reported that an increase in PN volume of 6.2 L (median) was required in 12 of 25 (48%) drug responders, and a further decrease in PS orders occurred in 7 patients (28%). Consistent with the results of the above study, BMI values in our sample remained stable over the 12 months (1 year). To the best of our knowledge, there are no other studies with an observation period longer than 12 months after treatment cessation to compare the results. 

According to Compher et al., a preserved colon, a longer small bowel, a lower baseline BMI, and a lower PS reduction during treatment predispose patients to a lower PS increase after treatment discontinuation. Jeppesen et. al. [17,32,33] reported that in patients with no colon, the beneficial effect of native GLP-2 wore off within a few weeks after treatment cessation. In contrast, in our study, the patient who maintained stable PS volume the longest—84 months (7 years) after treatment discontinuation—had an end jejunostomy (type I SBS) and a high (41%) on-drug reduction in PN volume. In addition, the highest PS increase within the first 12 months (1 year) after discontinuation of TED injections was reported in two patients with type II SBS and approximately 50% of the colon in continuity. However, because of the small number of patients and the homogeneity of the group (mainly patients with type II SBS), we did not perform an analysis comparing the value of changes in parenteral nutrition volume between patients with different types of short bowel syndrome. Further studies in larger groups of patients are needed to assess what factors contribute to the maintenance of stable PN volume after discontinuation of teduglutide.

The efficacy of teduglutide in reducing the need for parenteral support was confirmed in a recent systematic review and meta-analysis [34]. The authors analyzed a total of 10 studies, including both randomized controlled trials (RCT’s) and real-world retrospective and prospective cohort studies, and concluded that the response rate to teduglutide (proportion of patients with a reduction of ≥20% of PS volume) was 77% at 1 year and 82% at ≥2 years, with a significant increase between 6 months and 1 year of treatment (+29%, 95%CI: (+14%, +43%)), supporting the notion that the PS volume reduction likely increases with time after treatment initiation. The results of the STEPS-2 [35] and STEPS-3 [36] studies showed that long-term TED treatment (up to 3.5 years) was associated with sustained efficacy, as evidenced by a further reduction in PN volume, an increasing number of days without PN, and, in some patients, complete weaning from parenteral support in patients with SBS-IF. The rate of patients achieving full enteral autonomy (complete weaning from PS), which is a primary goal of TED treatment, varies from 15% to 92% in different reports. In a multicenter observational study, Joly et al. [24] reported 24% of patients weaned off PN after 24 weeks, whereas Puello et al. [36] demonstrated a 29% rate of enteral autonomy after a median follow-up of 3.2 years. These data are consistent with the findings of Iyer et al. [37] and Seidner et al. [38] and support the notion that complete weaning from PS is possible in a minority of treated patients. Nonetheless, the team of Harpain [39] reported a 92% rate of achieving enteral autonomy at a median follow-up of 107 weeks, with 23% of patients achieving enteral autonomy after 24 weeks of treatment. The authors emphasized the importance of individualized care and a patient-tailored approach by a multidisciplinary team of specialists. The available data suggest that teduglutide appears to be safe in patients with PN-dependent SBS, as most of the adverse effects reported were gastrointestinal in origin and were of mild or moderate severity, with abdominal pain being the most common adverse effect [35,40,41]. Another important question is the impact of TED therapy on the quality of life (QOL) of patients with SBS receiving parenteral nutrition. Although QOL of those patients is mostly related to the symptoms of the underlying disease, it has been shown that the reduction of PS as a result of treatment with TED can have positive effects on QOL [42], as PS infusions lasting up to 20 h per day can limit patients’ mobility, and both increased nocturnal urination and high stool frequency (or excessive stoma output) lead to sleep disturbances [43,44]. In addition, teduglutide was found to have a significant impact on quality of life in patients with underlying inflammatory bowel disease (IBD) and in patients with the highest baseline volume PS [45].

The results of our study suggest that in most cases, after an initial increase within the first year after treatment discontinuation (not statistically significant), the need for PN volume remains relatively stable for about 48 months (4 years) and then increases again 60–108 months (5–9 years) after treatment discontinuation. The rate of patients who required an increase in PN volume compared with the end of treatment values was 84.62% at 60 and 84 months (5 and 7 years) and 92.30% at 108 months (9 years). We reported that 108 months (9 years) after treatment discontinuation, the weekly PN volume exceeded baseline values by an average of 21.21% in 53.85% of patients. Analyzing the presented results in the context of the above-mentioned advantages of teduglutide treatment (in terms of safety, efficacy, possibility of discontinuation of HPN, and impact on the quality of life of SBS patients), the question arises whether further long-term or intermittent use of the drug would allow a further reduction or maintenance of stable parenteral nutrition volume or its complete cessation. However, treatment with teduglutide is very expensive, and data on the cost-effectiveness of the drug are lacking. 

It could be hypothesized that the reported increase in PN volume over time is not related to the previously received teduglutide treatment itself and that age or disease-related complications are the reason for the change in PN volume. On the basis of the analysis performed, we cannot exclude this possibility with certainty, however, the fact that all patients studied were physically active and independent throughout the observation period and were also able to participate independently in the follow-up examinations speaks against this hypothesis. Nevertheless, it is advisable to perform a comparative study with a control group of patients who received HPN for a similar period of time but never received teduglutide.

Our study has several limitations. Because it is a retrospective analysis, we analyzed only measures routinely performed in clinical practice during quarterly visits to HPN centers. Therefore, the analysis did not include measures of intestinal adaptation. In addition, the study group was quite small, making statistical conclusions difficult. Notwithstanding these limitations, the main strength of this study is the duration of observation. In addition, the analysis was based on real-life data, outside the boundaries of clinical trials, and therefore the results are more applicable to clinical practice. 

## 5. Conclusions

This is the first study to report changes in PN volume and BMI in patients with PN-dependent SBS after discontinuation of teduglutide treatment over an observation period of approximately a decade. The findings indicate that after an initial increase within the first 12 months (1 year) after stopping teduglutide, the PN volume remains relatively stable for approximately 4 years and increases again 60–108 months (5–9 years) after treatment discontinuation. The results also suggest that some patients require higher weekly PN volumes over time than before starting treatment with TED. The current report highlights the importance of closely monitoring the nutritional status and needs of patients with SBS who have discontinued treatment by a multidisciplinary team of qualified specialists. Further research is needed to determine what types of patients require the highest increase in PN volume and whether they would benefit from periodic use of teduglutide.

## Figures and Tables

**Table 1:** Characteristics of the study group.

	Female (*n* = 7)		Male (*n* = 6)		*z*	*p*-Value *	Total		
	Median (Range)	IQR/2	Median (Range)	IQR/2			Median (Range)	IQR/2	CV%
Age at T_0_ (y)	58.0 (26.0–78.0)	13.5	49.5 (25.0–73.0)	6.0	0.930	0.366	52.0 (25.0–78.0)	9.0	31.8
Age at T_end_ (y)	60.0 (28.0–80.0)	13.5	52.5 (28.0–75.0)	6.0	0.863	0.366	55.0 (28.0–80.0)	8.5	29.9
Predrug duration of HPN (mo.)	57.0 (20.0–127.0)	37.0	57.0 (20.0–161.0)	67.0	0.000	0.945	57.0 (20.0–161.0)	37.0	67.0
Duration of TED treatment (mo.)	30.0 (25.0–37.0)	0.5	36.0 (25.0–37.0)	3.0	−0.878	0.366	31.0 (25.0–37.0)	3.0	13.3

IQR/2: semi-quartile range, CV: coefficient of variation. * Mann–Whitney U test with continuity correction.

**Table 2:** Comparison of parenteral nutrition (PN) requirements and anthropometric measures before and after teduglutide treatment.

	Before Treatment (T_0_)				After Treatment (T_end_)				*z*	*p* *
	Mdn	IQR/2	Min	Max	Mdn	IQR/2	Min	Max		
PN Volume (ml/week)	10,680	3557.5	6680	18,900	6240	1990	3600	9345	3.180	0.001
PN Energy (kCal/week)	6050.0	2265.5	3258.5	13,258.0	5440.0	1996.0	2700.0	10,122.0	3.180	0.001
PN Amino acids (g/week)	212.50	90.00	104.13	437.50	170.00	56.25	89.25	437.50	2.521	0.012
Body weight (kg)	54.70	6.25	47.00	82.00	51.20	5.60	45.00	79.60	2.040	0.041
BMI (kg/m^2^)	21.45	1.31	18.51	26.47	19.97	0.66	17.72	25.70	2.197	0.028

Mdn: median, IQR/2: semi-quartile range. * Wilcoxon signed-rank test.

**Table 3:** Weekly parenteral nutrition (PN) volume in the study sample after stopping teduglutide.

	PN Volume (mL/Week)			
Time Point	Median	IQR/2	Min	Max
12 months off drug (T_+12_)	8270.00	2318.75	4575	15,120
24 months off drug (T_+24_)	8270.00	2537.00	5200	15,120
60 months off drug (T_+60_)	8305.00	1695.75	5200	15,610
84 months off drug (T_+84_)	8315.00	1960.00	4200	15,610
108 months off drug (T_+108_)	9400.00	2877.00	3150	22,610

IQR/2: semi-quartile range; Min: minimum; Max: maximum.

**Table 4:** Weekly parenteral nutrition (PN) energy and amino acid content in the study sample after stopping teduglutide (TED).

	PN Energy (kCal/Week)			PN Amino Acids (g/Week)		
Time Point	Median	IQR/2	Range	Median	IQR/2	Range
12 months off drug (T_+12_)	5950	2156	2760–12,229	212.50	111.25	102–437.5
24 months off drug (T_+24_)	5950	2156	3240–12,229	212.50	100.00	102–437.5
60 months off drug (T_+60_)	6500 *	1981	3240–12,712	250.00	90.00	102–437.5
84 months off drug (T_+84_)	6500 *	1631	2940–12,712	250.00	90.00	102–437.5
108 months off drug (T_+108_)	7245 *	1763	1935–13,636	297.50	93.75	93.75–437.5

IQR/2: semi-quartile range. * significantly different compared to Tend, *p* < 0.05 (post hoc Dunn–Šidák test).

## Data Availability

Data are available upon reasonable request to the corresponding author.
